# Early heart rate predicts 3-month outcomes in acute ischemic stroke patients receiving intravenous thrombolysis: a machine learning approach

**DOI:** 10.3389/fneur.2025.1668901

**Published:** 2025-09-09

**Authors:** Mian-Xuan Yao, Min-Yi Yao, Jia Gu, Ting Gao, Yi-Mian Yuan, Yang-Kun Chen, Yong-Lin Liu

**Affiliations:** ^1^Department of Neurology, The Tenth Affiliated Hospital of Southern Medical University (Dongguan People’s Hospital), Dongguan, China; ^2^School of Mathematics and Statistics, Huazhong University of Science and Technology, Wuhan, China; ^3^Center for Mathematical Sciences, Huazhong University of Science and Technology, Wuhan, China; ^4^Guangdong Provincial Key Laboratory of Mathematical and Neural Dynamical Systems, Great Bay University, Dongguan, China; ^5^First School of Clinical Medicine, Guangdong Medical University, Zhanjiang, China; ^6^Intelligent Brain Imaging and Brain Function Laboratory (Dongguan Key Laboratory), Dongguan People’s Hospital, Dongguan, China

**Keywords:** acute ischemic stroke, heart rate variability, intravenous thrombolysis, machine learning, prognosis

## Abstract

**Background:**

The predictive role of early heart rate (HR) dynamics in acute ischemic stroke patients (AIS) receiving intravenous thrombolysis (IVT) remains unclear. This study aimed to evaluate whether HR variability within 24 h post-IVT predicts early neurological deterioration (END) and 3-month functional outcomes using machine learning.

**Methods:**

This retrospective analysis included AIS patients without atrial fibrillation (AF) who received IVT at Dongguan People’s Hospital between January 2017 and December 2022. Hourly HR metrics (mean HR, SD, coefficient of variation [CV]) were analyzed. Primary outcomes were END (≥4-point NIHSS increase within 72 h) and poor 3-month outcomes (mRS ≥ 3). Machine learning models were developed and validated via receiver operating characteristic (ROC) analysis.

**Results:**

Among 381 patients, logistic regression identified NIHSS on admission (OR = 1.287, *p* < 0.001), maximum HR (OR = 0.956, *p* = 0.023), minimum HR (OR = 1.027, *p* = 0.001), and HR SD (OR = 1.356, *p* = 0.002) as independent predictors of poor 3-month outcomes. HR CV also showed significance but correlated strongly with SD. A machine learning model integrating onset-to-treatment time, NIHSS, and HR parameters (max/min HR, mean HR, SD) achieved an area under the ROC curve (AUC) of 0.82 for predicting 3-month outcomes. No HR metrics were significantly associated with END.

**Conclusion:**

In AIS patients without AF, early HR dynamics—particularly maximum HR, minimum HR, SD, and CV—strongly correlate with 3-month functional outcomes after IVT. The machine learning model demonstrated high predictive accuracy, highlighting the potential of real-time HR monitoring for risk stratification and personalized management in thrombolysis-treated AIS patients.

## Background

Acute ischemic stroke (AIS), which caused by sudden occlusion of cerebral vessels, is one of the major causes of disability and mortality globally and it is expected to increase in the coming years, due to the aging of the population (1). Therefore, early prediction of the functional prognosis of patients can help to carry out corresponding intervention as soon as possible. Intravenous thrombolysis therapy (IVT) is one of the most effective methods for improving functional outcomes following ischemic stroke. Previous studies have reported that outcomes after IVT are influenced by a variety of factors (2), including age (3), the National Institutes of Health Stroke Scale (NIHSS) score at admission, the Trial of Org 10,172 in Acute Stroke Treatment (TOAST) classification, and complications (4).

Some previous studies have revealed ANS dysfunction in patients after ischemic stroke (5, 6). Heart rate (HR) and heart rate variability (HRV) analysis, accurately representing the balance between the sympathetic and parasympathetic nervous systems and reflecting the overall stresses acting on the body, is a simple and non-invasive method to assess ANS function (7, 8). Currently, there are some studies on the relationship between HR and clinical prognosis after stroke. The study from Wang et al. (9), found that higher mean heart rate (MHR) and HRV were associated with the increased risk of 3-month all-cause mortality and worse functional outcome after mechanical thrombectomy therapy for AIS patients. Another study from Lee et al. (10), found that HR during the acute period of ischemic stroke (HR data between the 4th and 7th day after stroke onset) is a predictor of all-cause mortality during the first year after AIS. However, some of these studies only focused on a random HR after stroke, which cannot reflect the overall condition and changes of HR over a period of time. Some studies included patients with ischemic stroke of different pathogenesis, failing to reflect the impact of HR on the prognosis of these patients of different etiologies. In addition, some studies collected HR in different time periods, which may also affect results. Nowadays, studies focusing on the relationship between HR and outcome of AIS patients after IVT are still limited.

Random Forest (RF) is an ensemble learning method based on decision trees that has been widely applied in constructing clinical prognostic models in recent years. In the medical field, Random Forest is considered an effective tool for addressing big data analysis challenges due to its ability to handle high-dimensional data and complex non-linear relationships (11).

In the present study, we aimed to explore the influence of HR and HRV during the first 24 h after IVT on END and 3-month outcome in patients who received IVT and without atrial fibrillation (AF).

## Methods

### Patient selection

Consecutive patients with AIS who were treated with intravenous recombinant tissue plasminogen activator (r-tPA) and hospitalized at Dongguan People’s Hospital from January 1, 2017, to December 31, 2022, were enrolled in this study. The inclusion criteria encompassed: (1) individuals aged over 18 years; (2) AIS confirmed by magnetic resonance imaging (MRI) during hospitalization; (3) onset of ischemic stroke symptoms within 4.5 h prior to r-tPA administration; (4) continuous monitoring and hourly documentation of clinical features and heart rate (HR) for 24 h post-IVT; (5) a pre-stroke modified Rankin Scale (mRS) score of 1 or lower; and (6) comprehensive documentation, including follow-up data. Patients were excluded if they: (1) received additional endovascular therapy following IVT; (2) had AF; (3) were administered medications to control HR within 48 h before symptom onset or 24 h after IVT; or (4) were lost to follow-up. This study was granted approval by the ethics committee of Dongguan People’s Hospital (approval number: KYKT2024-063). Informed written consent was obtained from all the patients.

### Data collection

Demographic information, including age, gender, and medical histories of hypertension, diabetes mellitus, smoking, and previous strokes, was collected. Stroke subtypes were classified according to the TOAST criteria (12). Furthermore, the NIHSS score at admission, onset-to-treatment time (OTT), systolic blood pressure (SBP) at admission, and hourly HR recordings for 24 h after IVT were documented. SIASO was defined as a stenosis of at least 50% in the intracranial artery responsible for AIS (13). The degree of stenosis was measured using three-dimensional time-of-flight magnetic resonance angiography [MRI parameters are detailed in our previous publication (14)] by comparing the diameter of the stenotic vessel to the diameter of a normal vessel located distal to the stenosis (15).

### Definition of HRV

The HR data were collected as a standard part of patient monitoring during hospitalization, using an electrocardiogram monitor in the stroke units, and were then entered into the electronic medical records system. For all patients, HR was recorded hourly during the first 24 h after IVT. Eight patients underwent urgent CT examinations during the 24-h post-thrombolysis, resulting in isolated hourly gaps in their heart rate recordings. These missing values were imputed using linear interpolation of adjacent time-point measurements. We calculated the MHR and HRV in accordance with established guidelines (31). HRV was determined using the following formula (19):

(1) Standard deviation of MHR (SD):


$$\sqrt{1/(n-1)\sum_{i=1}^{\left(n-1\right)}{\left(\mathrm{HRi}-\text{HRmean}\right)}^{2}}$$

(2) Coefficient of variability (CV [%]): SD/HR_mean_ × 100 (16).

### Definition of clinical prognosis in the early stage

We used early neurological deterioration (END) as a measure to evaluate the clinical outcome of ischemic stroke in its initial stages. END is defined as a worsening of neurological status, indicated by an increase of ≥4 points in the NIHSS score within the first 72 h after admission (17).

### Definition of short-term clinical outcome

We conducted a 90-day follow-up with these patients to assess their mRS scores. A favorable outcome was defined as an mRS score of 2 or lower, whereas a poor outcome was defined as an mRS score of 3 or higher.

### Machine learning and feature filtering

Patients were randomly assigned to the training group and validation group at a ratio of 7:3. The random forest model quantified the importance of each feature. Using the random forest method, we explored the contribution of each clinical feature to 3-month poor outcomes and END.

### Statistical analysis

Continuous variables that exhibited a normal distribution were presented as the mean ± SD, whereas those that were non-normally distributed were reported as the median with the interquartile range. The statistics above were done using SPSS for Mac (version 26.0; IBM Corp., Armonk, NY, United States). Using Python (version 3.11.3), a random forest model was constructed. Logistic regression analysis was employed for both univariate and multivariate analyses to identify risk factors. The results were expressed as odds ratios (OR) and 95% confidence intervals (95% CI). In developing the clinical prediction model, variables with a *p*-value < 0.05 from univariate analysis were included in the multivariate analysis for variable selection. Variables with *p*-values < 0.05 in the multivariate analysis were used to construct the prediction model for END and adverse outcomes at 3 months. The accuracy of the model was evaluated using the ROC curve to visualize the AUC value. A *p*-value less than 0.05 was considered statistically significant.

## Results

### Patients’ characteristics

**Figure 1:**
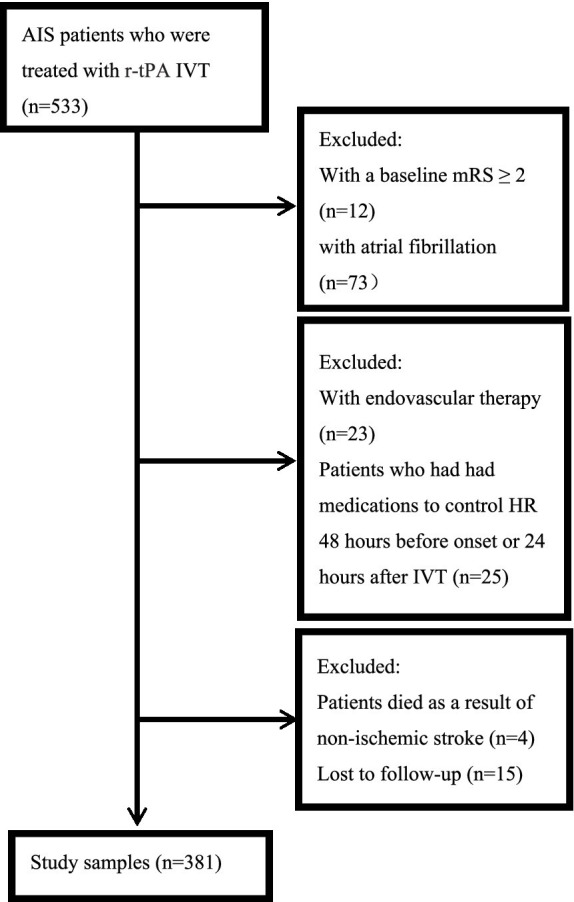
Flowchart of the selection process. AIS, acute ischemic stroke; IVT, intravenous thrombolysis; mRS, modified Rankin Scale; HR, heart rate.

A total of 533 patients with AIS who received IVT with r-tPA in our stroke unit were consecutively enrolled. However, 152 patients were excluded for various reasons: 12 had a baseline mRS score of ≥2, 73 had AF, 23 underwent additional endovascular therapy, 25 were taking medications to control HR within 48 h before onset or 24 h after IVT, 4 died from non-ischemic stroke-related causes, and 15 were lost to follow-up. Consequently, 381 patients were included in this study (Figure 1).

These 381 patients had a mean age of 61.1 ± 11.6 years. Among them, 291 patients (76.4%) were male, and 105 patients (27.5%) had stroke with SIASO. Thirty-one patients (8.1%) experienced END, and 77 patients (20.2%) had a poor outcome at the 3-month follow-up (Table 1).

**Table 1:** Patients characteristics of the study samples.

Characteristics	Total sample (*n* = 381)
Age^a^ (years)	61.1 (11.6)
Men^b^ (*n*,%)	291 (76.4%)
Hypertension^b^ (*n*,%)	270 (70.9%)
Diabetes mellitus^b^ (n,%)	98 (25.7%)
History of hypercholesterolemia^b^ (*n*, %)	160 (42.0%)
Smokers/ex-smokers^b^ (*n*, %)	160 (41.5%)
History of coronary heart disease^b^ (*n*, %)	26 (6.8%)
Previous stroke^b^ (*n*, %)	62 (16.3%)
SBP on admission^a^ (mmHg)	158.2 (25.5)
OTT^c^ (IQR, 25–75)	189.0 (136.0–240.0)
NIHSS on admission^c^ (IQR, 25–75)	5.0 (3.0–8.0)
SIASO^b^ (*n*, %)	105 (27.5%)
Maximum heart rate^a^ (bpm)	91.4 (14.6)
Minimum heart rate^a^ (bpm)	60.6 (9.7)
Mean heart rate^a^ (bpm)	72.8 (11.2)
SD^c^ (IQR, 25–75)	7.5 (6.0–9.3)
CV^c^ (IQR, 25–75)	10.4 (8.5–12.7)
END^b^ (*n*, %)	31 (8.1%)
mRS at 90 days^b^ (*n*, %)	
0	143 (37.5%)
1	100 (26.2%)
2	61 (16.0%)
3	39 (10.2%)
4	23 (6.0%)
5	9 (2.4%)
6	6 (1.6%)

^a^Mean (SD), *t*-test; ^b^*n* (%), chi-square test; ^c^Mann–Whiteny *U* test.

NIHSS, national institutes of health stroke scale; OTT, onset to treatment time; mRS, modified rankin scale; END, early neurological deterioration; SIASO, symptomatic intracranial artery stenosis or occlusion; SBP, systolic blood pressure; SD, standard deviation of heart rate; CV, coefficient of heart rate variation.

### Screening and ranking of important clinical features

**Figure 2:**
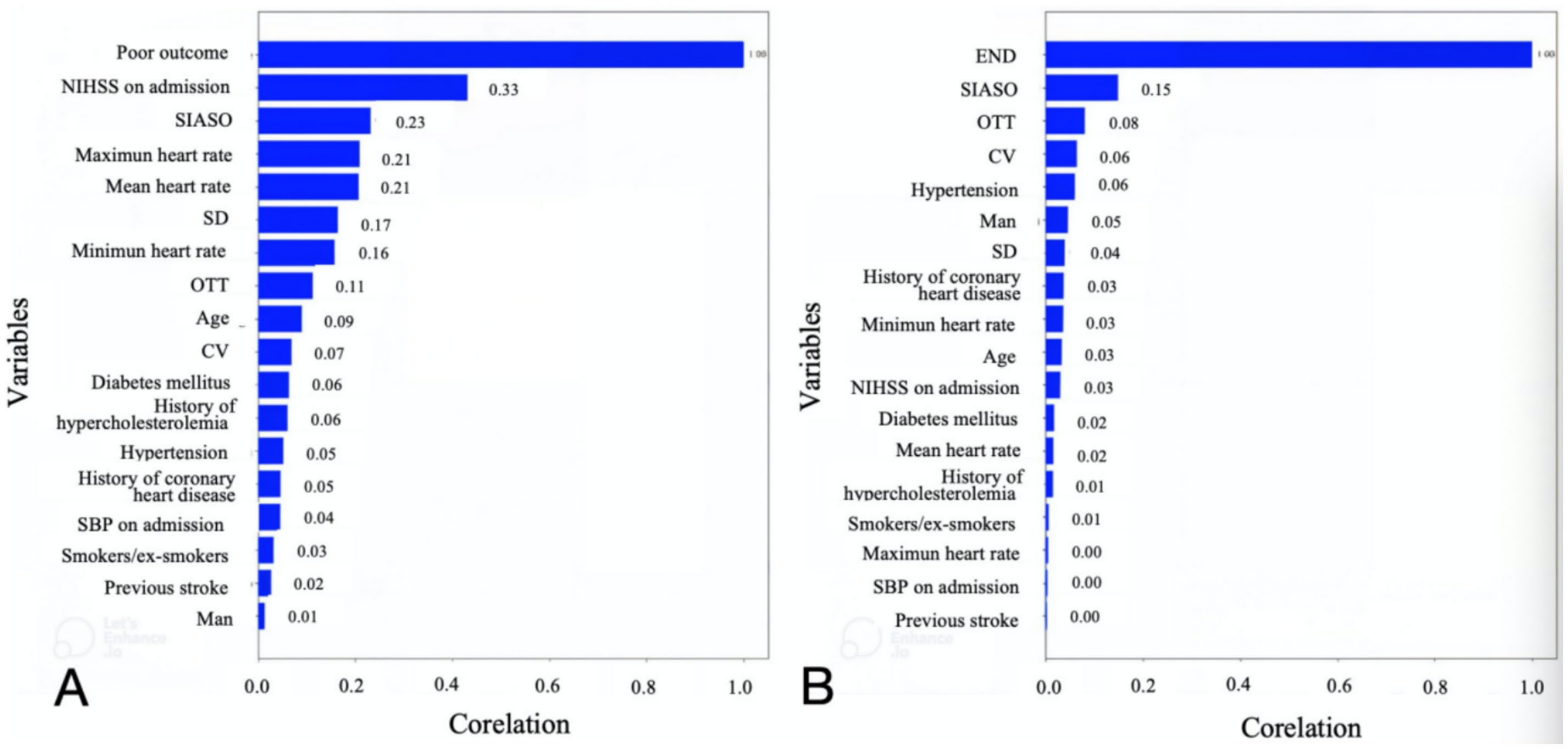
Plots of correlation of each variable with 3-momth poor outcome **(A)** and END **(B)**. NIHSS, national institutes of health stroke scale; OTT, onset to treatment time; END, early neurological deterioration; SIASO, symptomatic intracranial artery stenosis or occlusion; SBP, systolic blood pressure; SD, standard deviation of heart rate; CV, coefficient of heart rate variation.

**Figure 3:**
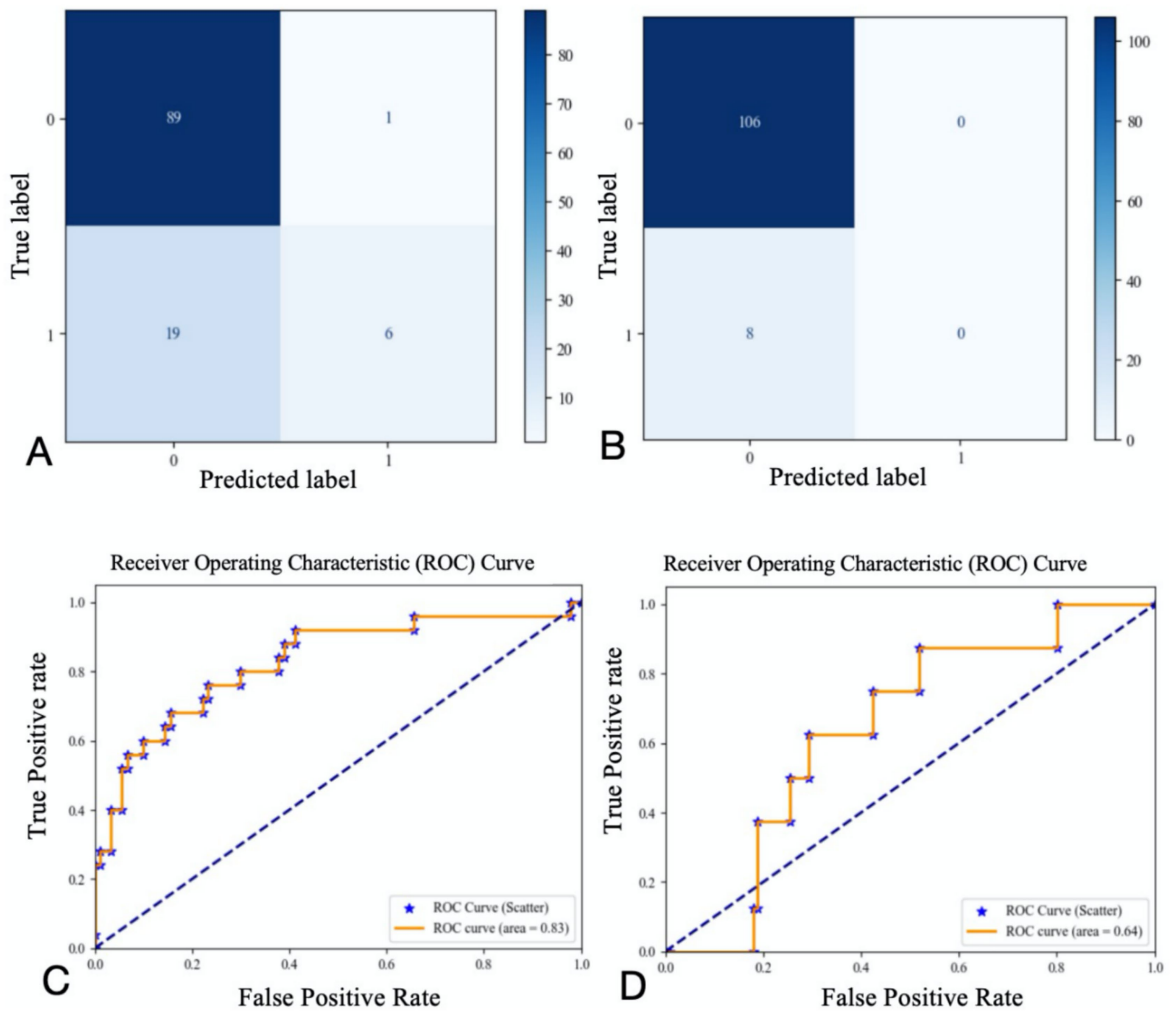
Confusion matrix and ROC curve for regression results (**A** confusion matrix for 3-momth poor outcome; **B** confusion matrix for END; **C** ROC curve for 3-momth poor outcome; **D** ROC curve for END).

**Figure 4:**
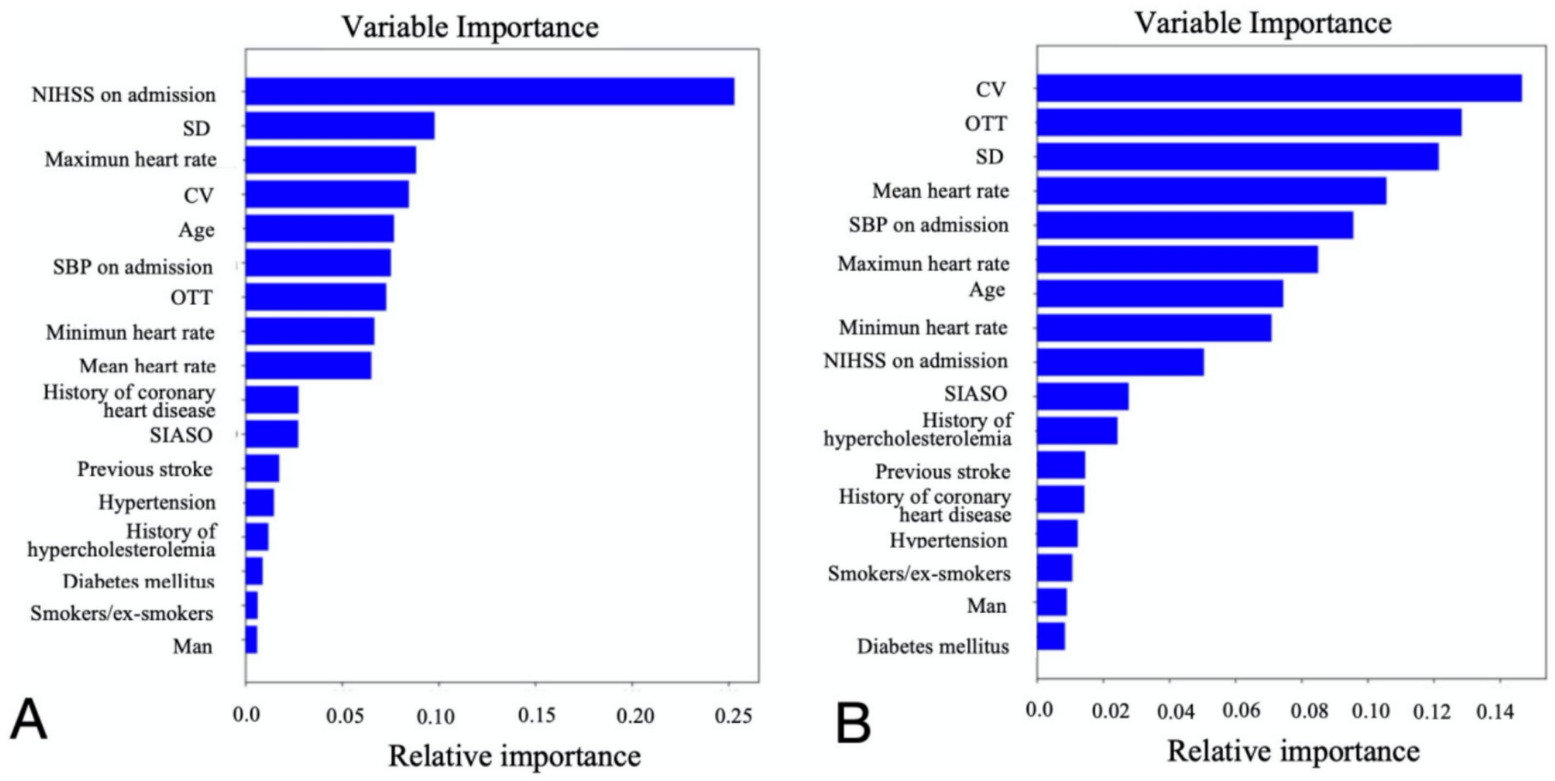
Relative importance of each variable for 3-month poor outcome **(A)** and END **(B)**. NIHSS, national institutes of health stroke scale; OTT, onset to treatment time; END, early neurological deterioration; SIASO, symptomatic intracranial artery stenosis or occlusion; SBP, systolic blood pressure; SD, standard deviation of heart rate; CV, coefficient of heart rate variation.

The study ultimately included 381 patients. Figure 2 illustrates the relationships between each independent variable and both 3-month poor outcomes and END. The correlation coefficients of all independent variables were evenly distributed. Using the random forest classification method, the 381 patients were randomly divided into a training set and a testing set in a 7:3 ratio. The training set was used to train the random forest classification model, while the testing set was used to predict 3-month poor outcomes and END. The results showed that the area under the ROC curve (AUC) for 3-month poor outcomes was 0.84, and for END, it was 0.64. The confusion matrix and ROC curve of the regression results are shown in Figure 3. The importance of each variable for 3-month poor outcomes and END was calculated and illustrated in Figure 4.

**Figure 5:**
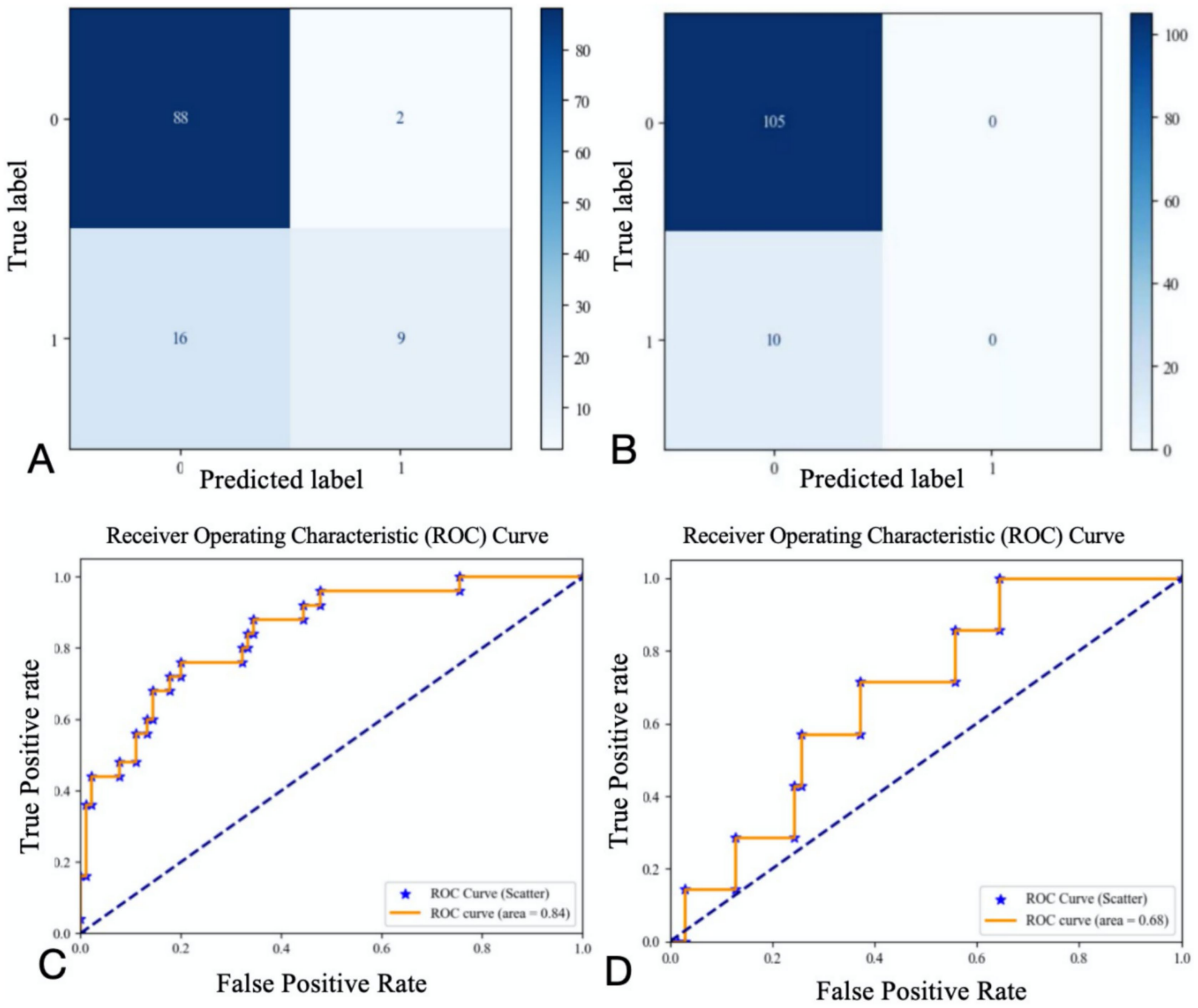
Confusion matrix and ROC curve of regression results for the first nine variables (**A** confusion matrix for 3-momth poor outcome; **B** confusion matrix for END; **C** ROC curve for 3-momth poor outcome; **D** ROC curve for END).

Based on the contribution of each variable to the outcomes, the top nine contributing factors were selected for analysis using the random forest classification model. These factors included NIHSS on admission, SBP on admission, OTT, age, SD, CV, maximum heart rate, minimum heart rate, and mean heart rate. Using 70% of the data as the training set and the remaining 30% as the testing set, the nine features were analyzed again for their impact on 3-month poor outcomes and END using the random forest classification model. The results showed that the AUC for the random forest classification model was 0.84 for 3-month poor outcomes and 0.68 for END. The corresponding confusion matrix and ROC curve are presented in Figure 5.

### Logistic regression model for predicting 3-month poor outcome

The SPSS was used to assess multicollinearity among the nine variables related to 3-month poor outcomes and END. It was found that SD and CV exhibited strong multicollinearity (VIF > 10, Table 2). Retaining both variables in statistical modeling would constitute redundant inclusion of a single underlying factor, thereby violating the independence assumption. Clinically, SD primarily reflects absolute safety thresholds of parameters (e.g., safe operating ranges for specific metrics), whereas CV captures intrinsic properties of biological variation (e.g., physiological fluctuation ranges). Given equivalent utility of SD and CV in HRV assessment and to ensure statistical robustness, we arbitrarily excluded one parameter—specifically CV—in this investigation.

**Table 2:** Collinear statistics of risk factors for 3-month poor outcome and END.

Variable	3-month poor outcome		END	
	Tolerance	VIT	Tolerance	VIT
Age	0.938	1.066	0.938	**1.066**
OTT	0.984	1.016	0.980	1.020
NIHSS on admission	0.960	1.042	0.945	1.058
SBP on admission	0.953	1.051	0.952	1.051
Maximum heart rate	0.108	9.279	0.107	9.318
Minimum heart rate	0.101	9.897	0.100	9.983
Mean heart rate	0.058	**17.209**	0.058	**17.373**
SD	0.022	**45.607**	0.022	**45.655**
CV	0.023	**43.881**	0.023	**44.090**

NIHSS, national institutes of health stroke scale; OTT, onset to treatment time; mRS, modified rankin scale; END, early neurological deterioration; SBP, systolic blood pressure; SD, standard deviation of heart rate; CV, coefficient of heart rate variation. Values in bold are statistically significant (*p* < 0.05).

A logistic regression model was used to analyze the individual effects of the remaining eight variables on 3-month poor outcomes. The results revealed that the following six variables had significant associations with 3-month poor outcomes (Table 3): OTT (OR = 1.004 [1.000–1.007], *p* = 0.039), NIHSS on admission (OR = 1.256 [1.179–1.338], *p* = 0.000), Maximum heart rate (OR = 1.034 [1.017–1.052], p = 0.000), Minimum heart rate (OR = 1.040 [1.014–1.066], *p* = 0.003), Mean heart rate (OR = 1.044 [1.022–1.068], *p* = 0.000), SD (OR = 1.167 [1.059–1.286], *p* = 0.002).

**Table 3:** Logistic regression of each risk factor for 3-month poor outcome and END.

Variable	3-month poor outcome			END		
	*β*	OR (95% CI)	*p-*value	*β*	OR (95% CI)	*p-*value
Age	0.019	1.020 (0.998–1.042)	0.082	0.013	1.013 (0.981–1.046)	0.432
OTT	0.004	1.004 (1.000–1.007)	**0.039**	0.004	1.004 (0.999–1.008)	0.094
NIHSS on admission	0.228	1.256 (1.179–1.338)	**0.000**	0.033	1.033 (0.960–1.112)	0.379
SBP on admission	−0.004	0.996 (0.986–1.006)	0.382	0.001	1.001 (0.987–1.016)	0.863
Maximum heart rate	0.034	1.034 (1.017–1.052)	**0.000**	0.001	1.001 (0.976–1.026)	0.950
Minimum heart rate	0.039	1.040 (1.014–1.066)	**0.003**	−0.010	0.990 (0.952–1.029)	0.603
Mean heart rate	0.043	1.044 (1.022–1.068)	**0.000**	−0.005	0.996 (0.963–1.029)	0.791
SD	0.155	1.167 (1.059–1.286)	**0.002**	0.070	1.072 (0.933–1.233)	0.326

NIHSS, national institutes of health stroke scale; OTT, onset to treatment time; mRS, modified rankin scale; END, early neurological deterioration; SBP, systolic blood pressure; SD, standard deviation of heart rate. Values in bold are statistically significant (*p* < 0.05).

**Figure 6:**
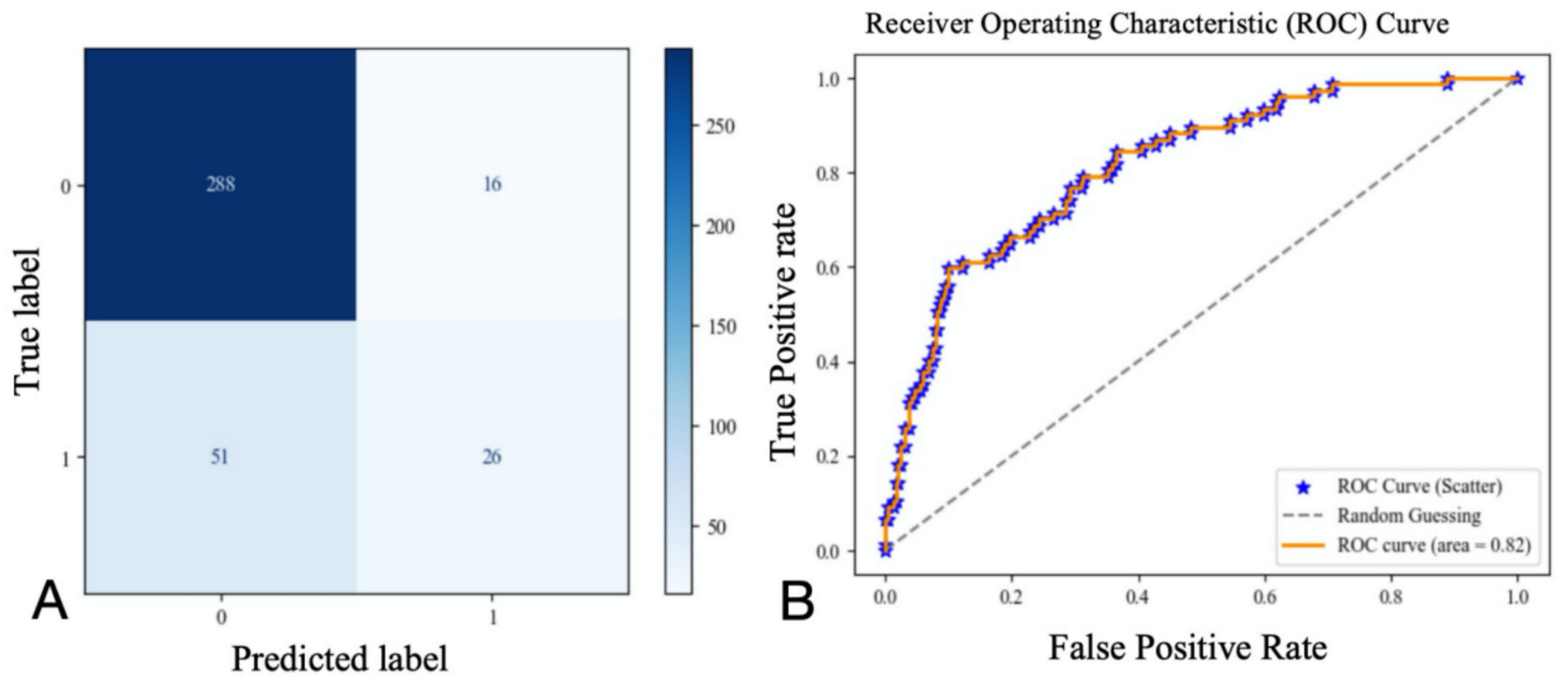
Confusion matrix **(A)** and ROC curve **(B)** of the regression results of the remaining six variables for 3-month poor prognosis.

A logistic regression model incorporating these six variables was used to analyze their combined impact on 3-month poor outcomes, with 70% of the data set as the training set and the remaining 30% as the testing set. The results showed that the AUC of the ROC curve was 0.82 (confusion matrix and ROC curve shown in Figure 6). Significant predictors for 3-month poor outcomes included: NIHSS on admission (OR = 1.287 [1.185–1.389], *p* = 0.000), SD (OR = 1.356 [1.248–1.464], *p* = 0.002), Minimum heart rate (OR = 1.027 [1.005–1.049], *p* = 0.001), Maximum heart rate (OR = 0.956 [0.945–0.967], *p* = 0.023). Given the strong multicollinearity between CV and SD, the impact of CV on 3-month poor outcomes was also significant (Table 4). Parameter setting of the random forest model was shown in Table 5.

**Table 4:** Logistic regression of all risk factors for 3-month poor outcome.

Variable	3-month		
	*β*	OR (95% CI)	*p-*value
OTT	0.004	1.002 (0.999–1.005)	0.541
NIHSS on admission	0.229	1.287 (1.185–1.389)	**0.000**
Maximum heart rate	−0.012	0.956 (0.945–0.967)	**0.023**
Minimum heart rate	0.006	1.027 (1.005–1.049)	**0.001**
Mean heart rate	0.031	1.036 (0.981–1.091)	0.488
SD	0.138	1.356 (1.248–1.464)	**0.002**

NIHSS, national institutes of health stroke scale; OTT, onset to treatment time; SD, standard deviation of heart rate. Values in bold are statistically significant (*p* < 0.05).

**Table 5:** Parameter setting of the random forest model.

Parameters	Set value
The number of decision trees (n_estimators)	500
The maximum tree depth (max_depth)	None
The splitting criterion	Gini impurity
The feature selection method	Square root of the number of features (sqrt)
The cross-validation strategy	Stratified 5-fold CV
Hyperparameter tuning	Randomized search (n_iter = 50)
The importance evaluation method	Permutation Importance

### Logistic regression model for predicting END

We also used a logistic regression model to analyze the effects of the remaining eight variables—age, OTT, NIHSS on admission, SBP on admission, maximum heart rate, minimum heart rate, mean heart rate, and SD—on END. The results showed that the *p*-values for all variables, including all heart rate-related variables in this study, were greater than 0.05 (Table 3; Supplementary material).

## Discussion

Our study demonstrates that maximum HR, minimum HR, HR CV, and HR SD within 24 h post-IVT are associated with 3-month unfavorable outcomes in non-AF AIS patients. Furthermore, we developed a clinical prediction model incorporating admission NIHSS, OTT, HR SD, maximum HR, minimum HR, and mean HR to predict 3-month unfavorable outcomes, which demonstrated high predictive value. However, HR and HRV parameters were not associated with END in our cohort. These findings may help clinicians identify non-AF AIS patients at elevated risk of poor 3-month outcomes, enabling timely interventions targeting HR and HRV control to potentially improve prognosis (18–20).

Our study suggests that higher HR SD and CV may predict poorer 3-month outcomes in IVT-treated patients without AF. HRV during the acute stroke phase has been associated with poor outcomes within the first 3 months post-ischemic stroke (21, 22). Nayani et al. (23) reported that patients with higher HRV had an increased likelihood of infarct expansion and poorer outcomes at 3 months and 1 year post-stroke (This study included first-ever ischemic stroke patients assessed via 24-h Holter for HRV within 2 to 4 weeks). Additionally, Tang SC et al. analyzed 1-h continuous ECG signals in acute stroke patients (both ischemic and hemorrhagic) admitted to the stroke ICU within 24 h; their findings indicate that early HRV assessment may help predict outcomes in non-AF patients (24). The study from Qu et al. (25) validated the prognostic value of HRV in thrombolysis patients without AF in their 2023 JAHA study, demonstrating significant associations with 3-month outcomes. Extending this framework, our research introduces methodological refinements through three targeted innovations to enhance prognostic assessment: (1) Temporal specificity: We focus on dynamic hourly HR metrics within the critical 24 h post-thrombolysis window, whereas Qu’s measurements at 1–3 and 7–10 days post-stroke may miss early autonomic dysregulation; (2) statistical methods: Our study employed a random forest classification method. This approach integrates multiple decision trees to effectively mitigate the risks of overfitting inherent in single decision trees while enhancing the model’s predictive accuracy; (3) clinical translation innovation: Our model relies solely on NIHSS and bedside monitor data without serological/imaging biomarkers, enabling real-time risk stratification – a significant advantage over Qu’s delayed HRV assessment.

Although the mechanisms through which HRV influences 3-month clinical outcomes in ischemic stroke patients remain incompletely elucidated, HRV constitutes a critical biomarker reflecting sympathetic-parasympathetic balance (7, 8, 26). It provides insights into cardiovascular autonomic regulation, where disruption of sympathetic-vagal equilibrium post-ischemic stroke induces alterations in HRV parameters (23, 27).

During acute ischemic stroke, ANS integrity significantly impacts 3-month prognosis through multiple pathways: Firstly, ANS-mediated vascular control may be impaired, potentially causing cerebrovascular dysregulation (e.g., vasospasm or pathological dilation), thereby compromising cerebral perfusion and neural function (28). Secondly, ANS dysfunction can disrupt neuroimmune interactions and impair neuromediator release, amplifying inflammatory cascades that exacerbate neuronal damage and hinder neural repair (29). Thirdly, post-stroke ANS disturbances contribute to metabolic dysregulation—including impaired glucose homeostasis and dyslipidemia—which potentiates secondary brain injury and impedes functional recovery (30, 31). Finally, such dysfunction is associated with neuropsychiatric sequelae (e.g., anxiety and depression), reducing rehabilitation adherence and compromising neurological recuperatio (32).

Our investigation revealed that elevated minimum and maximum HRs recorded within 24 h post-IVT correlated with poorer 3-month functional outcomes in non-AF stroke patients receiving IVT. This observation is substantiated by extant literature (9, 33, 34). Wang et al. (9) demonstrated that heightened HRs predict increased 3-month all-cause mortality and inferior functional recovery following mechanical thrombectomy in AIS patients. Complementary evidence from an AF-positive AIS cohort further established elevated HR—though not HR CV—as an independent predictor of 1-year mortality (33). Four interconnected pathophysiological pathways may elucidate this association: First, tachycardia likely signifies stroke-induced acute physiological stress, heralding adverse outcomes through neuroendocrine activation (34). Second, sympathetic overdrive elevates myocardial oxygen demand and arrhythmogenic susceptibility, compounding cardiac strain while reflecting ANS dysregulation (21, 26)—factors that collectively impair neurological recovery. Third, sustained tachycardia may indicate amplified inflammatory cascades post-stroke (35), wherein cytokine-mediated cardioneural injury and ANS dysfunction potentiate secondary brain damage via neurovascular uncoupling and immune dysregulation. Fourth, elevated heart rates may heighten hemorrhagic transformation (HT) risk, particularly in patients with cerebral microbleeds (CMBs) (36, 37), where intracranial hemorrhage directly exacerbates neurological deficits. Future mechanistic studies should delineate etiology-specific pathways and temporal dynamics governing heart rate-outcome relationships in ischemic stroke.

Our study developed a predictive model based on NIHSS score, CV, maximum heart rate, minimum HR, mean HR, and OTT, achieving an area under the ROC curve (AUC) of 0.82. This indicates that the model has high value in predicting 3-month poor outcomes in AIS patients undergoing intravenous thrombolysis. While the relationships between NIHSS on admission, OTT, and post-stroke clinical prognosis have been extensively reported, our study highlights the significant predictive value of heart rate and heart rate variability (HRV) for the 3-month clinical prognosis of patients treated with intravenous thrombolysis. The model does not rely on serological or imaging markers, allowing for a simple and rapid assessment of short-term prognosis. This facilitates early intervention for high-risk patients with poor outcomes, thereby improving clinical prognosis.

END is a serious clinical event following IVT in AIS, which may result from hemorrhagic or ischemic injuries (38). Although IVT represents the standard treatment for AIS, a subset of patients still experience END after therapy (39). HRV, serving as an indicator of autonomic nervous system function, has gained attention for its potential in predicting stroke outcomes (40). Although this study did not find a significant association between HR, HRV, and END, previous research has shown that HRV analysis can predict early neurological deterioration in stroke patients. A study by Nozoe et al. (21) found that increased sympathetic activity during the transition from a supine to a sitting position in stroke patients may be associated with END. The absence of a significant association between HR, HRV and END in this study may be attributed to the fact that HR and HRV primarily reflect the functional status of the ANS. These parameters may not adequately capture the complexity of the pathophysiological processes underlying stroke, particularly in patients with non-AF AIS (40). Post-stroke pathophysiological changes involve multiple factors, including inflammatory responses, cerebral edema, and neurotransmitter imbalances. These factors may exert indirect effects on HR and HRV, rather than being directly reflected by changes in these parameters (35). Secondly, the selected HR metrics measured at 24 h post-IVT may be insufficient to capture the relationship between HR/HRV and END (41). ANS after stroke represents a dynamic process, and alterations in HR and HRV may hold different implications at distinct phases following the event. Consequently, assessments conducted at varying time points could yield divergent results. Finally, the limited sample size—with only 31 patients experiencing END—combined with hourly heart rate measurements, may have influenced the study findings.

The random forest model developed in this study for predicting 3-month poor outcomes achieved an AUC of 0.82, demonstrating robust predictive value. However, we cannot rule out potential performance overestimation due to overfitting to cohort-specific patterns. Whether its predictive reliability remains comparable to our findings when applied to populations with different demographics, healthcare settings, or temporal contexts requires urgent validation through independent cohort studies to confirm the stability of these results. Despite inherent bias risks in the retrospective design, this study maximizes causal plausibility through strictly timed data acquisition (hourly HR measurements within 24 h post-thrombolysis) and prospectively adjudicated outcomes (90-day mRS). We contend the model’s clinical significance lies primarily in providing a rapid screening tool for resource-constrained settings (AUC 0.82), while also offering an important reference for future large-scale multicenter prospective studies.

This study has the following advantages: First, this study ranks among the few available investigations directly assessing the association between HR metrics and clinical prognosis in non-AF AIS patients receiving intravenous thrombolysis. Second, we collected heart rate data uniformly within the first 24 h after intravenous thrombolysis, avoiding biases caused by random sampling times. Finally, the study employed the random forest classification method, which integrates multiple decision trees to effectively reduce the risk of overfitting from individual trees and improve the predictive accuracy of the model.

There are several limitations in our study. First, the sample size was relatively small. Second, as a retrospective investigation, our study warrants validation through future large-scale, multicenter prospective studies to confirm these findings. Third, the limited occurrence of END in only 31 patients within the study sample may have influenced the result and a larger sample size is needed to confirm this result. Forth, since patients receiving endovascular therapy were excluded in this study, and these patients were all with SIASO, there may be bias in the samples of the SIASO group. Forth, the HR information was collected hourly, while long-term continuous dynamic HR recording may be needed. Finally, since laboratory parameters were not incorporated into the machine learning models, we cannot exclude the potential influence of these indicators on prognosis.

## Conclusion

Our study found that in non-AF AIS patients, maximum heart rate, minimum heart rate, HR SD, and HR CV within the first 24 h after IVT were significantly associated with 3-month poor outcomes. Using machine learning methods, we developed a predictive model for 3-month poor outcomes and found that this model demonstrated high predictive value. These findings suggest that early intervention targeting HR in AIS patients may help improve their clinical outcomes.

## Data Availability

The raw data supporting the conclusions of this article will be made available by the authors, without undue reservation.
