# Supplementary material for: Early heart rate predicts 3-month outcomes in acute ischemic stroke patients receiving intravenous thrombolysis: a machine learning approach
Source: Front Neurol. 2025 Sep 9;16:1668901. doi: 10.3389/fneur.2025.1668901 (PMC12454040; doi:10.3389/fneur.2025.1668901)
Supplement: Supplementary file 1 [file Data_Sheet_1.PDF]

Supplemental material

Table S1 | Biomarkers and medical history of the 3-month poor outcome in univariable analysis

	Favorable outcome (mRS=0-2) N=304	Poor outcome (mRS=3-6) N=77	*P* Value
Neutrophil-to-Lymphocyte Ratio (NLR)[c] (IQR,25-75)	2.96（2.23-4.81）	3.11 (2.32-5.71)	0.25
Glycated Hemoglobi (HbA1c)[a](%)	5.52(0.31)	5.72(0.46)	0.42
Low-Density Lipoprotein (LDL)[c] (IQR,25-75)(mmol/L)	2.25 (1.72-2.98)	2.42(1.63-3.21)	0.37
Homocysteine (Hcy)[a](umol/L)	14.51(3.52)	15.82(3.65)	0.49
History of coronary heart disease[b] (n,%)	19(6.3%)	7(9.1%)	0.38
Previous stroke[b] (n,%)	48(15.8%)	14(18.2%)	0.61

[a]*n (SD), t-test;* [b]*n (%), chi-square test;* [c]*Mann–Whiteny U test.*
